# Vaccination against Feline Panleukopenia: implications from a field study in kittens

**DOI:** 10.1186/1746-6148-8-62

**Published:** 2012-05-21

**Authors:** Verena Jakel, Klaus Cussler, Kay M Hanschmann, Uwe Truyen, Matthias König, Elisabeth Kamphuis, Karin Duchow

**Affiliations:** 1 Paul-Ehrlich-Institut, Paul-Ehrlich-Str. 51-59, 63225, Langen, Germany; 2 Institut für Tierhygiene und öffentliches Veterinärwesen, An den Tierkliniken 1, 04103, Leipzig, Germany; 3 Institut für Virologie, Fachbereich Veterinärmedizin (FB10) der Justus-Liebig-Universität Giessen, Schubertstr. 81, 35392, Giessen, Germany

## Abstract

**Background:**

Feline Panleukopenia (FPL) is a serious disease of cats that can be prevented by vaccination. Kittens are routinely vaccinated repeatedly during their first months of life. By this time maternally derived antibodies (MDA) can interfere with vaccination and inhibit the development of active immunity. The efficacy of primary vaccination under field conditions was questioned by frequent reports to the Paul-Ehrlich-Institut on outbreaks of FPL in vaccinated breeding catteries. We therefore initiated a field study to investigate the development of immunity in kittens during primary vaccination against FPL.

64 kittens from 16 litters were vaccinated against FPL at the age of 8, 12 and 16 weeks using three commercial polyvalent vaccines. Blood samples were taken before each vaccination and at the age of 20 weeks. Sera were tested for antibodies against Feline Panleukopenia Virus (FPV) by hemagglutination inhibition test and serum neutralisation assay in two independent diagnostic laboratories.

**Results:**

There was a good correlation between the results obtained in different laboratories and with different methods. Despite triple vaccination 36.7% of the kittens did not seroconvert. Even very low titres of MDA apparently inhibited the development of active immunity. The majority of kittens displayed significant titres of MDA at 8 and 12 weeks of age; in some animals MDA were still detected at 20 weeks of age. Interestingly, the vaccines tested differed significantly in their ability to overcome low levels of maternal immunity.

**Conclusions:**

In the given situation it is recommended to quantify antibodies against FPV in the serum of the queen or kittens before primary vaccination of kittens. The beginning of primary vaccination should be delayed until MDA titres have declined. Unprotected kittens that have been identified serologically should be revaccinated.

## Background

Feline Panleukopenia (FPL) is a contagious, serious disease of cats. The causative agent, Feline Panleukopenia Virus (FPV), belongs to the genus Parvovirus of the virus family Parvoviridae [1]. FPV is highly stable in the environment and endemic in many cat populations [2]. In a retrospective study FPL was identified as cause of death in 25% of kittens sent in for pathological examination [3]. The virus predominantly replicates in rapidly dividing cells. In newborn kittens, high mortality (>90%) with peracute deaths and neurological disorders like ataxia and blindness are the main clinical signs. Older kittens develop panleukopenia, neutropenia and diarrhoea due to infection of bone marrow, lymphatic tissue and intestinal epithelial cells. Clinical disease is most often diagnosed in kittens between 2 and 5 months of age while older cats predominately undergo subclinical or mild forms of the disease [2,4].

Vaccines against FPL are generally based on avirulent, replication competent virus strains (modified live vaccines, MLV). Successful vaccination induces virus-neutralising antibodies that provide protection against clinical FPL [2,5-7]. They can be used to monitor the immunological status of an animal [8]. In kittens lacking maternal antibodies, vaccination with MLV leads to seroconversion with high and long-lasting antibody titres [9,10]. Queens can transfer maternal antibodies mainly via colostrum conferring protection against disease during the first weeks of life [8,11]. The individual status of maternal immunity has been reported to differ considerably between kittens and is dependent on various factors such as the volume of colostrum ingested, the quality of the colostrum and the time of nursing [12]. Maternally derived antibodies (MDA) interfere with the development of active immunity after vaccination. Kittens with MDA titres ≥1:10 measured by hemagglutination inhibition (HI) did not show seroconversion after vaccination with a MLV [8]. Therefore, vaccination is not recommended before the level of MDA has fallen below this value. After the complete disappearance of MDA a single vaccination with MLV may lead to seroconversion and high, long-lasting titres [13].

While low titres of MDA do not generally provide protection against infection, they may still interfere with vaccination [8]. The period between the disappearance of protection by maternally derived passive immunity and the ability to respond to vaccination with the development of active immunity is referred to as “critical phase” or “immunological gap”. During this period, a high incidence of severe cases of FPL is observed [4]. Several attempts have been made to determine the optimal vaccination age for kittens in order to shorten the time at risk. Scott et al. suggested calculating the time point, when MDA have fallen to a level allowing successful vaccination, based on the antibody titre of the queen [8]. Other authors suggested testing the offspring prior to vaccination [14]. In the latter study performed in dogs a significant correlation between the antibody titres of littermates was observed.

Due to the lack of responsiveness in kittens with MDA most manufacturers recommend double vaccination against FPL at an interval of 3 to 4 weeks starting at the age of 6–8 weeks for primary immunisation. In any case, the last dose should be given beyond 12 weeks of age. Some manufacturers suggest postponing primary vaccination until 12 weeks of age if high MDA titres are expected. In this case, application of the second dose is recommended at 16 to 20 weeks of age [15].

Current recommendations on vaccination against FPL have been questioned by reports showing that protective immunity is not achieved in a significant proportion of cats. Dawson et al. observed a high number of kittens without antibody response at the age of 15 weeks after two or three vaccinations (25 and 39% respectively) [16]. Similar results were obtained in other studies [17,18]. Reports of severe disease in vaccinated animals indicate the clinical significance of non-responding cats and dogs [4,19,20].

Since 2007 several outbreaks of FPL in vaccinated breeding catteries of Norwegian Forest Cats (NFC) were reported to the Paul-Ehrlich-Institut. The incidents were treated as cases of suspected lack of expected efficacy (SLEE). Subsequently we initiated a field study to investigate the serological immune response of NFC after vaccination against FPL. Kittens were vaccinated three times at four week intervals with commercially available FPL vaccines as recommended for primary vaccination by the “German Standing Vaccination Committee Vet” [21]. The development of antibody titres against FPV was monitored in all animals until the age of 20 weeks.

The hemagglutination inhibition (HI) test is widely used to determine the antibody titre against FPV [22-24]. Since no international standard has been established so far for any method the comparison of results from different laboratories and test methods is difficult [25]. We therefore decided to combine the vaccination efficacy study with a comparison of test methods and laboratory performance.

## Results

### Correlation of test results

All serum samples were tested in two diagnostic laboratories using two different methods, respectively. The inter-laboratory variation and predictive values showed a good correlation between methods (HI vs SNT: Lab A 0.983 Lab B 0.804) and laboratories (Lab A vs Lab B: HI 0.897 SNT 0.949). In general, low responding or high titre sera were classified identical by both test methods and laboratories. Nevertheless, different cut-off values discriminating negative from low titre sera had to be considered.

**Figure 1:**
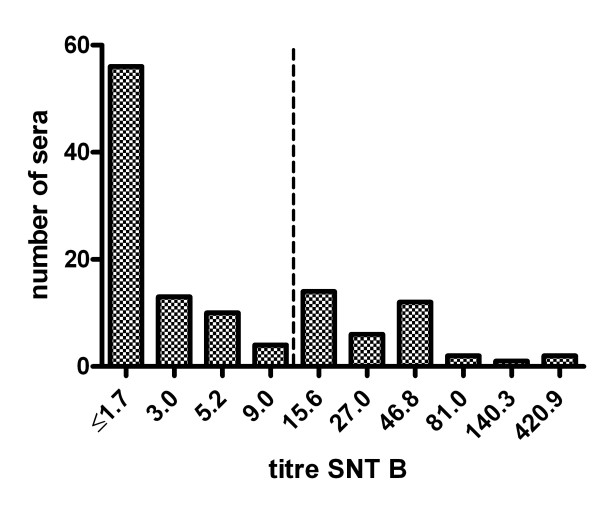
**Test results of HI A negative sera (<1:10) by SNT B.** The inverse titre is shown. The vertical dotted line indicates the arbitrary titre of 1:10 below which interference of MDA with vaccination might not be expected.

Laboratory A reported negative results for approximately half of the samples. In the majority of these sera, low titres of neutralising antibodies were detected by SNT B (Figure 1), indicating a higher sensitivity of this method.

For statistical analysis the results of 248 blood samples from 62 kittens were available. Two kittens were excluded from statistical analysis (Table 1): one kitten (NFC) showed clinical signs of disease on the day of the first vaccination; for the other kitten (European House Cat - EHC) no follow-up was possible.

**Table 1:** Overview on the number of litters and animals per group

	**NFC**		**EHC**		
					
**Vaccination groups**	**Number of litters**	**Number of kittens**	**Number of litters**	**Number of kittens**	****Total (kittens)****
I	3	14	2	10	**24**
II	3	15	2	6*	**21**
III	4	12*	2	7	**19**
**Total**	**10**	**41**	**6**	**23**	**64**

* Missing results led to the exclusion of one kitten in this group from the statistical analyses. NFC = Norwegian Forest Cats, EHC = European House Cats.

When antibody titre results of the two test groups (NFC and EHC) were compared, no statistically significant differences in the humoral immune response were observed (*p* >0.05). As a consequence the differentiation between breeds was dismissed for the remaining analyses.

### Maternal antibodies can persist for extended periods of time

All queens tested (12/15) had high antibody titres (SNT B; range from 1:46.8 to 1:34092) before birth or on the day of the first or second vaccination of the kittens.

**Figure 2:**
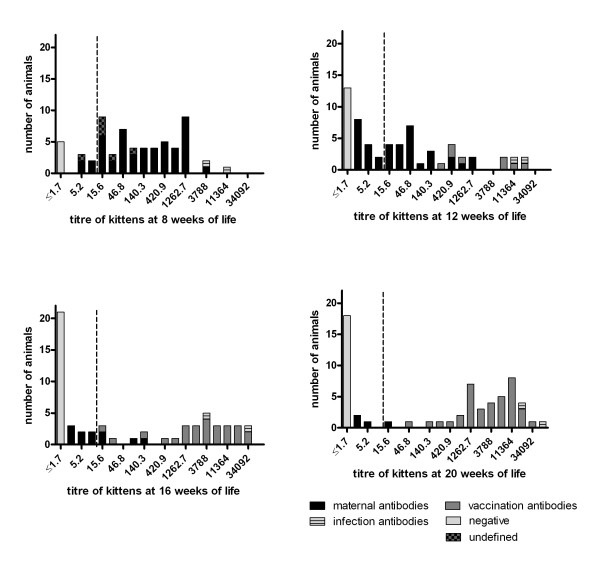
**Development of antibody titres against FPV in kittens during primary vaccination measured by SNT B.** The inverse titre is shown. The vertical dotted line indicates the arbitrary titre of 1:10 below which interference of MDA with vaccination might not be expected. All kittens were vaccinated three times with 8, 12 and 16 weeks of age. Allocation to the respective group (maternal, infection, vaccination antibodies) was based on the antibody course in the individual animal as explained in the text. In some kittens differentiation between active and passive immunity at 8 weeks was unclear and the antibodies titled as “undefined”.

**Figure 3:**
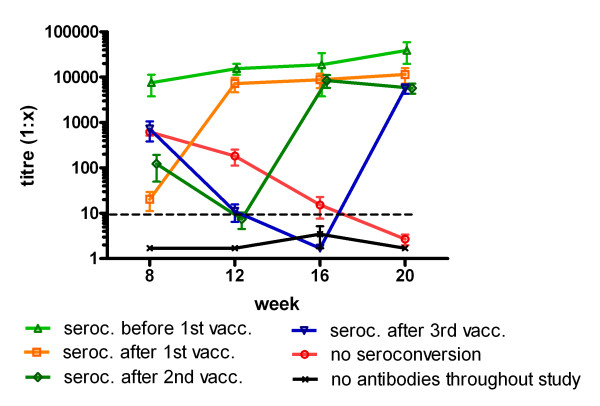
**FPV antibody titres and the time point of seroconversion in kittens measured by SNT B.** The mean titre and the SEM are shown. The horizontal dotted line indicates the arbitrary titre of 1:10 below which interference of MDA with vaccination might not be expected.

When the kittens were tested prior to the first vaccination at 8 weeks of age 92% of them had antibodies against FPV (Figure 2 and Figure 3). Detected antibody titres can either represent MDA or be the result of an early infection with FPV. For differentiation the course of antibody titres during the study was evaluated in each individual animal.

Six kittens showed a significant increase in antibody titres after the first vaccination. The remaining kittens showed decreasing antibody titres after first vaccination indicating maternal antibodies. In a high number of animals, antibody titres further decreased after the second and even after the third vaccination indicating MDA as well. Overall, MDA were observed in many kittens at 8 and 12 weeks of age (Figure 2) and in 4 kittens at 20 weeks of age. The calculated half-life of MDA was 8.8 days on average (litter range 6–11 d).

Two littermates had high antibody titres >1:1000 (SNT B) already before the first vaccination remaining at this level during the time of the study (Figure 2 and Figure 3). Apparently, these kittens had been exposed to natural infection.

In two littermates from a litter of three kittens, no antibodies were detected during the entire study.

### Homogenous reaction patterns between littermates

Reaction patterns between littermates were generally similar. In 10 of the 16 litters all kittens seroconverted following the same vaccination and in 5 litters the kittens reacted to consecutive vaccinations (data not shown). When titres were compared at 8 weeks of age there was low variability between littermates with differences of up to 1:2 in 11 litters (72%), 1:4 in three and 1:16 in one litter (HI A).

### Absence of seroconversion in a high number of kittens despite repeated vaccinations

In our study seroconversion did not occur in 36.7% of the kittens despite triple vaccination (Figure 2). There was a relationship between the presence of MDA and the probability of seroconversion. After seroconversion antibody titres remained relatively constant at high levels in all kittens.

Despite the absence of detectable HI antibodies (HI A; <1:10), no seroconversion was found in 70% of the samples (data not shown); 37% of the kittens had titres <1:10 twice and 33% three or four times in a row. When the results of SNT B were analysed, the number of animals that did not seroconvert despite the absence of detectable antibodies (1:1.7) dropped to 49% with 25% of the kittens consecutively reacting negative twice. This indicates that even minimal antibody titres beyond the detection level of the assays are capable to inhibit seroconversion.

### Correlation between the antibody titre of the queen and seroconversion rate of kittens

**Figure 4:**
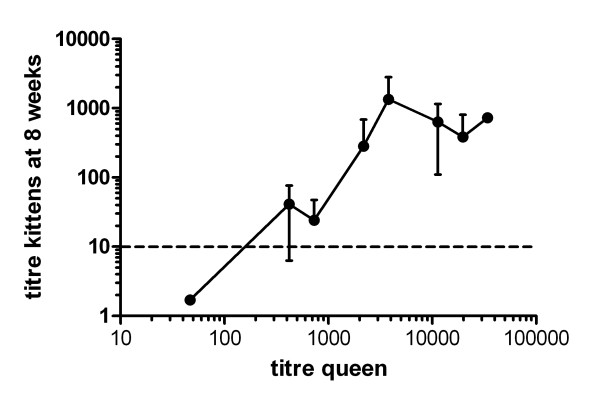
**Relationship between the antibody titres of the queen and their kittens measured by SNT B 8 weeks after birth**. The horizontal dotted line indicates the arbitrary titre of 1:10 below which interference of MDA with vaccination might not be expected.

The antibody levels of the queens correlated with the MDA titre of the respective kittens (Figure 4) as well as with the vaccination performance. Queens with low antibody titres had a higher percentage of successfully immunised kittens showing seroconversion at earlier time points (Table 2). 14 out of 16 kittens from queens with antibody titres <1:1000 (SNT B) seroconverted during the study: 25% after the first vaccination and 56% after the second vaccination. In contrast no seroconversion was observed in the majority of kittens (17/23) from queens with antibody titres >1:10000. Two kittens from a queen with a high antibody titre (1:3788) showed rising antibody titres after the first vaccination. The reaction of these animals may have been the result of natural infection seen in this litter (Table 2).

**Table 2:** Effect of the queens’ antibody titre on vaccination results of the kittens

**antibody titre of the queen**	**<1000**		**1000–<10000**		**>10000**	
seroconversion of kittens:	**N**	**%**	**N**	**%**	**N**	**%**
after first vaccination	4	**25**	0	**0**	2*	**9**
after second vaccination	9	**56**	5	**36**	0	**0**
after third vaccination	1	**6**	6	**43**	4	**17**
no seroconversion	2	**13**	3	**21**	17	**74**
**Total**	**16**		**14**		**23**	

*In this litter the third littermate died from FPL by the time of first vaccination.

### Calculation of starting points avoids unnecessary vaccinations

The apparent correlation between antibody titres of queens and the response of their kittens to vaccination allows improvement of the vaccination schedule. Along this line we calculated the starting point for primary vaccination based on antibody titres of the queen determined by SNT B. The starting point was defined as the calculated age when MDA will reach the titre value of 1:10.

68% of the kittens that seroconverted during the study showed a serological response after vaccination at the calculated starting point or later (data not shown). The response rate using a modified vaccination scheme including first vaccination at or after the calculated starting point followed by revaccination 4 weeks later was comparable to the triple vaccination scheme.

Similar results were obtained when the calculation of the starting point was based on individual antibody titres of kittens. 50% of kittens that seroconverted responded to a vaccination given when the calculated titre of MDA was ≤1:10. Based on our data we set up the respective equation for calculation as follows:

(1) $$a=age^{*}+8.8\frac{ln(titre^{**})-ln(b)}{ln(2)}$$

*in days of blood sampling

**on day of blood sampling

a = age of the kitten when its MDA titre will reach the specified titre “b”

In order to determine the success of vaccination discrimination between actively induced antibodies and MDA is essential. In animals older than 12 weeks, high titres (>1:1000) measured by both test systems could be assigned to active immunity after seroconversion in nearly all cases (Figure 2). With antibody titres below 1:1000 differentiation between MDA and active immunity was not feasible, even beyond 12 weeks of age.

### Significant influence of vaccines

In our study we used three different commercially available vaccines based on replication competent FPV. The statistical analysis of SNT results demonstrated significant differences between vaccines of different manufacturers (Lab A: *p* = 0.0388; Lab B: *p* = 0.0066). Virtually all animals with repeated minimum titre results after vaccination belonged to group I. In this group, a negative serum titre (1:1.7, SNT B) was predictive for seroconversion after a following vaccination only in one third of the cases. In contrast a negative result was followed by seroconversion in virtually all cases in groups II and III.

In some animals seroconversion occurred despite titres >1:10. All respective animals belonged to vaccination group II.

## Discussion

The aim of the current study was to evaluate the efficacy of vaccines against Feline Panleukopenia (FPL) under field conditions. The indicator chosen was the development of antibody titres after vaccination, which is a commonly used indirect method. FPL is a serious health risk especially to young cats. Neutralising antibodies, passively acquired or due to active immunity, are known to confer protection against the disease [2,5-8]. Maternally derived antibodies (MDA) interfere with the development of active immunity after vaccination [8,11,25,26]. In a study based on one vaccine Scott came to the conclusion that by 12 weeks of age MDA titres have declined to levels that are overcome by MLV [27]. Our data demonstrate that this assumption should not be generalized. We found antibody titres likely to represent MDA in most kittens until 12 weeks of age and beyond. As a consequence, only 45% of the kittens were successfully immunised by a twofold vaccination at 8 and at 12 weeks, representing the immunisation scheme recommended for primary vaccination by most manufacturers. Even application of a third vaccine dose at 16 weeks of age left one out of three kittens unprotected. Our findings are in line with results from previous field studies. Dawson et al. showed that only 75% of kittens seroconverted after multiple vaccinations until 12 weeks of age [16]. Similar results were reported in recent studies [17,18]. The lack of successful immunisation following the recommended vaccination scheme explains the occurrence of clinical disease in the post-weaning period both in vaccinated as well as non-vaccinated animals [4]. The inability to induce a significant serological response (i.e. seroconversion) implicates complete neutralisation of the vaccine virus. Consequently priming effects leading to an enhanced serological response after revaccination as postulated by Poulet as well as the induction of cell mediated immunity cannot be expected [17]. This was demonstrated by the high percentage of kittens requiring three vaccinations until seroconversion and the large proportion that failed to seroconvert despite triple vaccination. Furthermore it has been shown that kittens and puppies are susceptible to parvovirus infection after disappearance of passive immunity in spite of previous infections with virulent FPV or CPV during the phase of protection by MDA [8,28]. Along this line vaccinations with MLV that do not induce seroconversion must be regarded as needless. It must be assumed that the animals remain susceptible to FPV infection despite vaccination.

We found that in some animals even without detectable levels of antibodies vaccination was unsuccessful. Similar observations have been reported from studies in puppies [28]. The reason for the vaccination failure observed in the latter animals is not clear. Levels of interfering antibodies below the detection limit of the test systems may be responsible. Interestingly we observed significant differences between individual vaccines in their performance in MDA-positive kittens (*p* <0.05). Seroconversion in the presence of MDA titres >1:10 was only observed in animals from vaccination group II.

Our study was designed to evaluate the immune response in different cat breeds and between littermates. The number of litters in each group was therefore limited. Since the statistical model considered the litter size, results with regard to effects of different vaccines are reliable. Further field studies should address the efficacy of different vaccines during primary vaccination of young kittens.

When littermates were compared with regard to their antibody titres and their ability to respond to immunisation our results partly support previous studies. Friedrich found a high correlation between the HI- antibody titres of littermates in a study in dogs including 58 litters [14]. 97% of the puppies had HI-antibody titres that differed from the titres of their littermates not more than a single titre step (dilution 1:2). The variability of antibody titres in kittens of the same litter observed in our study was slightly higher. Nevertheless, the reaction pattern within a litter was similar: in 2/3 of the litters seroconversion was induced in all kittens following the same vaccination. Apparently littermates are susceptible to vaccination at similar time points allowing generalisation of test results of individual animals within a litter.

In addition we found a close relationship between antibody titres of the queens and the kinetics of MDA titres in the respective kittens which is in line with previous reports [8].

Our results demonstrate that the current recommendations on primary vaccination of kittens should be revised. Titres of MDA in kittens mainly depend on the immunological status of their queen. In homogenous litters with good colostrum supply similar maternally derived immunity can be expected. The majority of kittens in our study were from middle-aged vaccinated breeding cats and displayed relatively high MDA titres. In contrast, Dawson et al. found a high percentage of kittens from rescue shelters serologically negative at the age of 6 weeks [16]. For optimal vaccination results the primary vaccination scheme should be adapted to the situation of the individual kitten or litter. We propose to start vaccination at the age when kittens show low MDA titres corresponding to the ability to raise an active immune response. In previous publications the critical MDA titre was determined as ≤1:10 [8]. The results of our study generally confirm this assumption showing that about two-thirds of kittens will develop an active immunity after single vaccination at this starting point or thereafter. Therefore, we recommend measuring the antibody titre either of the queen or of kittens to calculate the starting point for vaccination. Identification and revaccination of unprotected animals by individual post-vaccination efficiency control is another key factor to increase the success rate of vaccination against FPL. It should be noted however that titre values refer to specific test systems and standardisation between methods is generally missing.

There were some immanent limitations of our study. Monitoring of infection status was not performed. This might have led to overestimation of vaccination efficacy if seroconversion was induced by natural infection instead of vaccination. Challenge infections to test immunity were not performed. Therefore only indirect conclusions on vaccination efficacy based on the development of the antibody titres can be drawn. Despite these limitations we would like to stress that the current study is the first containing independently collected data to assess the vaccination efficacy of FPL vaccines in the field.

## Conclusions

Our study revealed serious problems associated with primary vaccination against FPL. A significant proportion of kittens failed to develop active immunity following the recommended vaccination scheme.

The results help to explain the high incidence of clinical disease despite good vaccination coverage. Additional studies on the performance of different vaccines in the presence of MDA in combination with epidemiologic data on disease incidence and vaccine breakdowns are necessary for final adaptation of the vaccination recommendations. The epidemiologic situation may be improved by the development of new generation vaccines that overcome passive immunity more easily. Meanwhile, adjustment of the starting point for primary vaccination to the individual situation of each kitten supported by serology is one approach to reduce the incidence of FPL.

## Methods

### Animals

#### Queens

If possible, a serum sample from the queen was obtained approximately 1 week before giving birth and on the day of the first vaccination of the kittens (8 weeks after birth). Samples from 12 queens were available for analysis. In one queen the sample was taken on the day of the second vaccination of the kittens (12 weeks after birth).

### Kittens

Breeders and owners of NFC and European house cats (EHC) were recruited. In two litters of EHC the breeding history was unknown and the queen was not available. Complete litters of each breed were assigned to one of three vaccination groups (I, II and III). The numbers of litters and animals per group are indicated in Table 1.

### Vaccinations

Kittens were vaccinated at 8, 12 and 16 weeks of age using three commercially available polyvalent life vaccines against FPL and cat flu (RC - rhinotracheitis, calicivirus infection). Upon request of the owners, vaccines against chlamydiosis, feline leukaemia and rabies were also given. Vaccinations were performed following the product information supplied by the manufacturers. The vaccine ranges used were Nobivac from Intervet, Purevax from Merial and Virbagen felis from Virbac (in alphabetical order).

### Blood sampling

A blood sample was taken from each kitten immediately prior to vaccinations at 8 (pre-vaccination control), 12 and 16 weeks of age. A final blood sample was obtained at 20 weeks of age. The sera were prepared by centrifugation, labelled individually with a serial number and stored at −20°C until testing. The sera of the queens were treated accordingly. After receipt of the last sample, the sera were thawed and aliquoted. The sample numbers were randomized and blinded. One aliquot of each serum was sent to two independent diagnostic laboratories (Laboratory A and Laboratory B).

### Hemagglutination inhibition (HI) tests

The HI test was performed in both laboratories according to the same protocol. The sera were diluted in PBS and pretreated by heat inactivation (30 min, 56°C) followed by incubation with 12.5% (w/v) kaolin suspension for 20 min at room temperature. After removal of kaolin by centrifugation the supernatant was pre-adsorbed to swine erythrocytes for 1 h at 37°C and subsequently diluted in borate buffer (BBS pH 9.0) over twelve steps in a geometrical 1:2 series starting at 1:10 in 96-well round bottom plates (Greiner, Frickenhausen) [29]. To each well 4–8 hemagglutinating units of feline parvovirus (strain 292, provided by U.Truyen Leipzig) in BBS were added and incubated for 1 h at 18–25°C. After cooling of the plates to 4°C a 1% suspension of pig erythrocytes in phosphate buffer (0.15 M NaCl 0.3 M NaH2PO4, pH 4.5) was added. Following an incubation period of 90–120 min at 4°C the plates were read macroscopically. The HI titer was given as the highest dilution of serum showing complete inhibition of hemagglutination. Control reactions without virus antigen were used to exclude nonspecific effects.

### Serumneutralisation tests (SNT)

#### Laboratory A

For the titration of neutralising antibodies heat inactivated sera were prediluted 1:10 followed by duplicate geometrical 1:2 dilution series prepared directly in 96-well plates. To each well 100 TCID_50_ of FPV (strain 292) were added and incubated for 1 h at 37°C. After addition of indicator cells (CRFK, ATCC CCL-94) the plates were incubated for 5 days at 37°C in a humified cell culture incubator. Reading of the test results was done by indirect immune fluorescence using monoclonal antibodies followed by goat anti-mouse FITC-conjugated IgG (Dianova, Hamburg) [30]. The titre was given as the last dilution step with complete neutralisation.

#### Laboratory B

The test was essentially performed as in Laboratory A with minor changes: (i) sera were diluted in a geometrical 1:3 series and (ii) antibodies used for immune fluorescence staining were CPV1-2A1 (Acris, Herford) as first antibody followed by goat anti-mouse H + L conjugated to Cy3 (Dianova, Hamburg). Titres were calculated according to the method of Spearman and Kärber.

#### Interpretation of test results

Interpretation of test results was performed in accordance with the guidelines supplied by the test laboratories. For the HI test antibody titres ≥1:10 were regarded as positive by both test laboratories. Different cut off values were used for the two SN assays: Laboratory A ≥1:10 and Laboratory B >1:1.7. A difference of antibody titres in consecutive sera of the same animal by a factor of at least 4 (HI and SNT A) or 5.2 (SNT B) was regarded as significant, i.e. demonstrating seroconversion.

#### Statistical analysis

The statistical analysis was performed separately for each laboratory (A, B) and the two methods (HI, SNT) for the logarithmic titres in a mixed-linear model for repeated measurements. The fixed factors were *time* (week of blood sampling after birth), *breed* (NFC, EHC), *vaccine* (I, II or III) and *interaction between breed and vaccine*. As random factors *litter* and *animal* (repeated measures) were included in the calculation. P-values and confidence intervals were calculated and α-adjusted by the method of Bonferroni. Values of *p* <0.05 were considered significant.

The half-life of maternal antibodies was calculated as described by Scott et al. [8]. For calculation of the earliest time point for successful vaccination based on the antibody titre of the queen, the equation set up by Scott et al. was used: “x = 4.5*(q-y), where x is the age of the kitten at which the maternally derived titre of this kitten will reach the specified -log value y and q is the -log titre of the queen” [8].

## Authors’ contributions

VJ designed the study, coordinated data collection, was responsible for interpretation of results and drafted the manuscript. KC conceived of the study and made substantial contributions to its conception and design. KMH participated in the design of the study and performed the statistical analysis. UT was responsible for laboratory diagnostics and made substantial contributions to the drafting of the manuscript. MK was responsible for laboratory diagnostics and made substantial contributions to the analysis and interpretation of data and drafting of the manuscript. EK made substantial contributions to the analysis and interpretation of data and drafting of the manuscript. KD made substantial contributions to the conceptual design of the study and gave final approval of the version to be published. All authors read and approved the final manuscript.
